# Sleep characteristics and problems of 2-year-olds with Williams syndrome: relations with language and behavior

**DOI:** 10.1186/s11689-020-09336-z

**Published:** 2020-11-20

**Authors:** Caroline Greiner de Magalhães, Louise M. O’Brien, Carolyn B. Mervis

**Affiliations:** 1 Department of Psychological and Brain Sciences, University of Louisville, 317 Life Sciences Building, Louisville, KY 40204 USA; 2 Sleep Disorders Center, Department of Neurology, University of Michigan, Ann Arbor, USA

**Keywords:** Behavior problems, Language development, Neurodevelopmental disorders, Pediatrics, Sleep problems, Sleep-related breathing disorders, Sleepiness, Williams syndrome

## Abstract

**Background:**

Sleep problems have been shown to have a negative impact on language development and behavior for both typically developing children and children with a range of neurodevelopmental disorders. The relation of sleep characteristics and problems to language and behavior for children with Williams syndrome (WS) is unclear. The goal of this study was to address these relations for 2-year-olds with WS. Associations of nonverbal reasoning ability, nighttime sleep duration, and excessive daytime sleepiness with language ability and behavior problems were considered.

**Method:**

Ninety-six 2-year-olds with genetically confirmed classic-length WS deletions participated. Parents completed the Pediatric Sleep Questionnaire, which includes a Sleep-Related Breathing Disorder (SRBD) scale with a subscale measuring excessive daytime sleepiness, to assess sleep characteristics and problems. Parents also completed the Child Behavior Checklist (CBCL) and the MacArthur-Bates Communicative Development Inventory: Words and Sentences to assess behavior problems and expressive vocabulary, respectively. Children completed the Mullen Scales of Early Learning to measure nonverbal reasoning and language abilities.

**Results:**

Parents indicated that children slept an average of 10.36 h per night (*SD* = 1.09, range 7.3–13.3), not differing significantly from the mean reported by Bell and Zimmerman (2010) for typically developing toddlers (*p* = .787). Sixteen percent of participants screened positive for SRBD and 30% for excessive daytime sleepiness. Children who screened positive for SRBD had significantly more behavior problems on all CBCL scales than children who screened negative. Children with excessive daytime sleepiness had significantly more attention/hyperactivity, stress, and externalizing problems than those who did not have daytime sleepiness. Individual differences in parent-reported nighttime sleep duration and directly measured nonverbal reasoning abilities accounted for unique variance in expressive language, receptive language, and internalizing problems. Individual differences in parent-reported daytime sleepiness accounted for unique variance in externalizing problems.

**Conclusions:**

The relations of nighttime sleep duration, positive screens for SRBD, and excessive daytime sleepiness to language and behavior in toddlers with WS parallel prior findings for typically developing toddlers. These results highlight the importance of screening young children with WS for sleep problems. Studies investigating the efficacy of behavioral strategies for improving sleep in children with WS are warranted.

**Supplementary Information:**

The online version contains supplementary material available at 10.1186/s11689-020-09336-z.

## Background

Young children’s sleep problems impact not only their daytime behavior [1] but also their parents’ physical health, stress, and quality of life [2]. Negative outcomes of sleep problems for typically developing (TD) children include language delay [3–5] and behavior problems [1]. Sleep problems are even more common among young children with neurodevelopmental disorders [6–10] and also have been associated with both more limited language ability [11, 12] and behavior problems [13–15].

In the present study, we considered the sleep characteristics of a relatively large sample of 2-year-olds with Williams syndrome (WS), a neurodevelopmental disorder with a prevalence of 1 in 7500 [16] live births caused by a microdeletion of 26–28 genes on chromosome 7q11.23 [17], based on parent report on a validated sleep questionnaire. We also examined the relations between the children’s sleep characteristics and their language development and behavior problems. The WS cognitive phenotype is characterized by mild to moderate developmental delay or intellectual disability including relative strengths in concrete language and nonverbal reasoning and considerable weakness in visuospatial construction [18, 19]. Almost all children with WS evidence language delay, ranging from mild to severe [20, 21]. More than half of children with WS meet DSM-IV criteria for an anxiety disorder (primarily specific phobia) and more than 60% meet DSM-IV criteria for ADHD [22]. The prevalence of clinically significant social, anxiety, and attention problems as reported by parents of children with WS on the Child Behavior Checklist (CBCL) [23] is considerably higher than for the CBCL norming sample [24].

### Association of child sleep characteristics and problems to language and behavior

For young TD children, longer nighttime sleep duration is associated with better language development [3–5], although the effect size usually is small. Smithson et al. [3] found that TD 24-month-olds with long nighttime sleep had significantly higher composite language scores on an examiner-administered standardized assessment than those with intermediate or short nighttime sleep. Lam et al. [4] reported that for TD preschoolers, nighttime sleep duration was significantly but weakly positively associated with receptive vocabulary ability as measured by an examiner-administered standardized assessment. Touchette et al. [5] found that children who consistently slept less than 10 h per night between the ages of 2.5–6 years were significantly more likely than children who consistently slept 10 or more hours per night to score at least 1 *SD* below the population mean on an examiner-administered standardized assessment of receptive vocabulary at age 5 years. For young children with Down syndrome, nighttime sleep duration was significantly positively associated with parent-reported receptive vocabulary size but not with parent-reported expressive vocabulary size [11], and young children with Down syndrome who had at least 80% sleep efficiency had significantly larger parent-reported expressive vocabularies than children with lower sleep efficiency [12].

Sleep characteristics also are associated with behavior in young children. For TD children, shorter nighttime sleep duration is associated with difficulties in emotion regulation [25], and both reduced nighttime sleep duration [5, 26] and excessive daytime sleepiness [27] have been associated with externalizing problems. There is consistent evidence of an association between sleep disturbance and ADHD symptoms [28–32]. Although the association of sleep characteristics with behavior problems in young children with neurodevelopmental disorders is less often studied, published studies consistently identify problems. For example, for children with intellectual disability of mixed etiology, sleep problems were associated with externalizing behavior problems [13], and for children with autism spectrum disorder, shorter nighttime sleep duration was associated both with increased parent-reported behavior problems as measured by the CBCL [14] and with greater difficulty in social interaction and higher overall diagnostic characteristics of autism [15].

In the present study, we also were interested in Sleep-Related Breathing Disorder (SRBD), a spectrum of breathing disturbances ranging from habitual snoring to obstructive sleep apnea with a prevalence of 1–10% for the general pediatric population [33]. Tamana et al. [34] found that TD 2-year-olds who screened positive for SRBD (as measured by the SRBD scale from the Pediatric Sleep Questionnaire, PSQ [35]) are at greater risk for internalizing behavioral problems. Smithson et al. [3] found that TD 24-month-olds with persistent sleep-disordered breathing symptoms from age 3 months to age 24 months scored significantly lower on an examiner-administered standardized language assessment than children who did not have persistent symptoms. In addition, TD 4–10-year-olds with SRBD have lower cognitive/language abilities than children who do not have SRBD [36, 37]. Furthermore, TD 5–7-year-olds with primary snoring are rated by their parents as having more attention problems, social problems, and anxious/depressive symptoms and score lower on some measures of language and visuospatial abilities relative to children without a history of snoring [38]. More generally, a meta-analysis indicated that TD children with SRBD have more attention deficit/hyperactivity symptomatology than do TD children who do not have SRBD [39]. Similar results have been reported for children with Down syndrome who have SRBD [40].

### Sleep characteristics and problems in children with Williams syndrome

Studies addressing sleep characteristics and problems of individuals with WS have primarily used parent report measures. In the only study that focused on infants (mean age 20 months), the proportion of parents who reported that their child had sleep problems was similar to that for parents of similarly aged TD controls [10]. Nighttime sleep duration for full-term infants with WS did not differ significantly from that for TD controls [10]. Beyond infancy, findings have consistently indicated that children with WS have more sleep problems than age-matched TD children, including sleep anxiety, bedtime resistance, sleep onset delay, frequent night waking, general restlessness, and excessive daytime sleepiness [10, 11, 41–47]. Findings regarding nighttime sleep duration are less consistent with some authors reporting no significant differences in total time slept [42–44, 46] and others reporting that children with WS sleep less on average than same-aged TD children [10, 11, 47].

In keeping with caregiver reports of increased sleep difficulties, children with WS evidence increased bedtime cortisol and a less pronounced rise in melatonin levels relative to TD children [48, 49]. Overnight polysomnography findings have indicated that individuals with WS had decreased sleep efficiency, increased respiratory-related arousals, increased slow wave sleep [41], increased non-rapid eye movement percentage, and decreased rapid eye movement sleep percentage and irregular sleep cycles relative to TD controls [50, 51].

Two small-sample studies have examined the relation between parent-reported nighttime sleep duration and parent-reported vocabulary size in young children with WS. Axelsson et al. [47], in a study of 14 children aged 18–48 months, found a significant positive concurrent effect between nighttime sleep duration and expressive vocabulary size as measured by the MacArthur Communicative Development Inventory: Words and Gestures [52] (*p* = .021) after taking into account the linear contribution of chronological age. However, the concurrent nonlinear effect of chronological age, which would be expected to be considerable for vocabulary growth over this age range [53, 54], was not considered, making the finding difficult to interpret. D’Souza et al. [11], in a study of 30 children aged 9–52 months using the Oxford University version of the MacArthur Communicative Development Inventory: Words and Gestures, found a significant concurrent effect of nighttime sleep duration on receptive vocabulary size (*p* = .048) but not expressive vocabulary size (*p* = .061) after taking the concurrent nonlinear effect of chronological age into consideration. The authors note that if there had been any correction for multiple comparisons, neither effect would have been significant.

Relations between behavior and various sleep variables for children with WS were considered in two prior studies with relatively small sample sizes. Mason et al. [41] found no significant differences between children with WS who had ADHD features and those who did not on a variety of sleep measures. Axelsson et al. [47] found no significant differences in nighttime sleep duration between children who scored in the normal range on the CBCL—1.5–5 [23] Emotional Reactivity and Attention Problem scales and those who scored in the borderline or clinical range on these scales.

### Current study

Previous studies of sleep in children with WS have included relatively small samples spread over relatively broad age ranges and compared the children with WS to relatively small samples of TD children. In the present study, we took a different approach, focusing on a considerably larger sample (*N* = 96) of children with WS over a very narrow age range (24–35 months). This strategy allowed us to provide a detailed overview of sleep characteristics for 2-year-olds with WS as reported by their parents on a well-validated questionnaire, the PSQ [35], and to evaluate possible relations of sleep characteristics to language abilities and behavior problems. We also compared parent-reported nighttime sleep duration for the present sample to the parent-reported nighttime sleep duration for TD toddlers in a large previously published sample [55]. Based on prior literature for TD children [5, 26, 34, 36, 37], we expected that toddlers with WS who screened positive on the SRBD scale or had excessive daytime sleepiness would have more limited language abilities and more behavior problems than those who screened negative. In addition, based on prior literature regarding TD children [25], we expected that nighttime sleep duration would account for a significant amount of variance in individual differences in behavior problems and, based on prior literature regarding both TD children [4] and the two small studies of children with WS [11, 47], we expected that nighttime sleep duration would account for a significant amount of variance in individual differences in expressive and receptive language, although the effect sizes were expected to be small. Based on the TD literature [26, 27] and the neurodevelopmental disorders literature (e.g., [56]), we also expected that excessive daytime sleepiness would account for a significant amount of variance in externalizing problems.

## Method

### Participants

Children were included in this study if they had a genetically confirmed classic-length deletion of the WS region, were between the ages of 2.00 and 2.99 years, and did not also have another syndrome associated with intellectual disability. The final sample included 96 toddlers (40 girls, 56 boys) with genetically confirmed classic-length WS deletions ranging in age from 2.00 to 2.98 years (*M* = 2.31 years, *SD* = 0.31, *Mdn* = 2.16). The racial/ethnic distribution was 83 White non-Hispanic (86.5%), three White Hispanic (3.1%), two Asian non-Hispanic (2.1%), seven biracial non-Hispanic (7.2%), and one biracial Hispanic (1%). English was the native language of all 96 participants; six participants also had some exposure to another language. Participants were from 31 states in the USA (including all four US census districts) and the District of Columbia. One additional 2-year-old with a genetically confirmed classic WS deletion was excluded from the final sample because he also had a chromosome 15q13.3 deletion. Data collection began in February 2006 and ended in June 2019.

### Measures

#### Pediatric Sleep Questionnaire (PSQ)

The PSQ [35] is a validated gold standard parent report measure of sleep problems in children aged 2–18 years. It includes a 22-item SRBD scale which has three subscales: Snoring (4 items), Sleepiness (4 items), and Behavior (6 items). For most items, response choices are no/yes/do not know. Items addressing symptoms of inattention or hyperactivity are rated on a 4-point Likert scale that was converted to a no (0 or 1)/yes (2 or 3) scale. Chervin et al. [35] considered a child to have SRBD if the parent answered at least 33% of the items affirmatively. Parents of all participants responded “yes” to the item regarding nocturnal enuresis, which is developmentally appropriate for 2-year-olds. Thus, this item was excluded from the SRBD scale and a child was considered to have a positive screen for SRBD if s/he received at least 7 of the remaining 21 points. For the Snoring and Sleepiness subscales, a child was considered to screen positive if s/he received at least one point (at least one yes response). For the Behavior subscale, a child was considered to screen positive if s/he received at least two points (at least two yes responses). Chervin et al. [35] showed that the SRBD scale and its three subscales had high reliability and validity when compared to polysomnography objective criteria and concluded that the PSQ was an appropriate measure to use when polysomnography was not practical.

The PSQ also includes questions addressing other sleep characteristics such as difficulty falling asleep at night, difficult bedtime routines, or taking medication for sleep. These responses were analyzed to provide descriptive information regarding the sample. Parent responses to PSQ questions regarding when the child goes to bed at night, when s/he wakes up in the morning, and how long it takes her/him to fall asleep were used to calculate how many hours the child slept per night on average (nighttime sleep duration).

#### Child Behavior Checklist for ages 1.5–5 (CBCL)

The CBCL [23] is a parent report measure of problem behaviors. Each behavior is rated on a 0 (not true), 1 (somewhat or sometimes true), or 2 (very true or often true) scale. The CBCL yields three higher-order factor scales: Internalizing Problems, Externalizing Problems, and Total Problems. We also considered parent ratings on the five Diagnostic and Statistical Manual of Mental Disorders-5 (DSM-5)-based scales and on the Stress Problems and Sleep Problems scales. *T* scores range from 28 (Externalizing, Total) or 29 (Internalizing) to 100 on the higher-order scales and from 50 to 100 on the other scales.

#### Mullen Scales of Early Learning (MSEL)

The MSEL [56] is a standardized assessment of early cognitive and language abilities for very young children. For the present study, performance on the Visual Reception (measuring primarily nonverbal reasoning), Expressive Language, and Receptive Language scales was considered. Performance on the Fine Motor scale was not included because of extremely limited variability; 49 of the 96 participants (51%) earned the lowest possible *T* score. For the general population, the mean *T* score for each scale is 50 (*SD* = 10, range 20–80).

#### MacArthur-Bates Communicative Development Inventory: Words and Sentences 2nd edition [CDI]

The CDI [57] is a parent report measure addressing children’s vocabulary and grammatical abilities. The 680-item Vocabulary Checklist from the CDI: Words and Sentences form was used as a measure of the child’s expressive vocabulary (EV). Parents were given detailed instructions [21] to ensure that they only included words that their child both understood and said/signed spontaneously. The child’s EV was the total number of words the parent marked as says/signs. To facilitate comparisons involving children of different ages, Mervis et al. [58] normed the CDI Expressive Vocabulary Checklist for children with WS aged 18–48 months in 1-month intervals [mean standard score (SS) = 100, *SD* = 15]. The concurrent validity of CDI-EV SS for the present sample of children was excellent, as indicated by the correlation of CDI-EV SS with MSEL Expressive Language *T* score, *r* = .88.

### Study procedures

This study was approved by the University of Louisville’s Institutional Review Board, and parents/legal guardians of all participants provided written informed consent. All measures were administered according to the standardized procedures. The PSQ and CBCL were mailed to parents a few days in advance of their child’s assessment and parents were asked to bring the completed forms with them. On the day of the assessment, parents filled out the CDI with one of the researchers while a second researcher administered the MSEL to the child. The parent report measures were completed by the child’s mother or by both parents together.

### Statistical analyses

Data were analyzed using IBM SPSS v. 25. Due to uneven sample sizes, nonparametric Mann-Whitney *U* tests were performed to compare the group of children who screened positive on the SRBD scale to the remaining children. The same procedure was used to compare the group of children who screened positive on each of the SRBD subscales to the children who screened negative. Using the program provided by Lenhard and Lenhard [59], Cohen’s *d* was calculated as a measure of effect size (0.20 = small effect, 0.50 = medium, 0.80 = large). The Holm-Bonferroni correction for multiple comparisons was used to ensure that the target *α* value of *p* < .05, 2-tailed, was maintained.

Pearson correlations were used to compute bivariate relations between the sleep measures and the language, nonverbal reasoning, and behavior problems measures. For the correlational analyses, *α* was set at *p* < .01, 2-tailed. Two sets of five linear multiple regressions were performed with the language variables CDI-EV SS, MSEL Expressive Language *T*, and MSEL Receptive Language *T* and the behavior variables CBCL Internalizing Problems *T* and CBCL Externalizing Problems *T* as the dependent variables. For the first set, MSEL Visual Reception *T* and nighttime sleep duration were mean-centered and entered as the predictors. For the second set, MSEL Visual Reception *T* and sleepiness score were mean-centered and entered as the predictors. All linear multiple regression assumptions were met and no outliers were identified. All independent variables were entered at the same time. Cohen’s *f*^2^ was used to measure effect size (0.02 = small effect, 0.15 = medium, 0.35 = large). The Holm-Bonferroni correction for multiple comparisons was used for the multiple regression analyses to ensure that the target *α* value of *p* < .05, 2-tailed, was maintained.

## Results

### Full sample: descriptive statistics

Descriptive statistics for the PSQ SRBD scale and its subscales are presented in Table 1. Of the 15 children (15.6%) who screened positive on the SRBD scale, 10 were boys and five were girls. For the full sample, descriptive statistics are reported in Table 2 for sleep latency, nighttime sleep duration (number of hours in bed minus amount of time to fall asleep), and CBCL *T* scores and in Table S1 for CDI-EV SS and MSEL *T* scores.

**Table 1:** Descriptive statistics for the Pediatric Sleep Questionnaire SRBD scale and subscales

SRBD scale or subscale	*N*	Maximum possible score	*M* (SD)	Mdn (IQR)	Range	*N* positive (%)
SRBD^b^	96	21^a^	3.74 (2.72)	3.00 (2.00–5.75)	0–11	15 (15.6%)
Snoring	92	4	0.41 (0.76)	0 (0–1)	0–3	26 (28.3%)
Sleepiness	70	4	0.51 (0.93)	0 (0–1)	0–4	21 (30.0%)
Behavior	96	6	1.99 (1.65)	2 (1–3)	0–6	75 (78.1%)

*SRBD* Sleep-Related Breathing Disorder, *IQR* interquartile range

^a^Score of 7 or higher on the SRBD scale, 1 or higher on the Snoring and Sleepiness subscales, and 2 or higher on the Behavior subscale

^b^The 22nd item, measuring nocturnal enuresis, was excluded because nocturnal enuresis is developmentally appropriate for 2-year-olds

**Table 2:** Descriptive statistics for the full sample (*N* = 96) for sleep latency, nighttime sleep duration, CDI-EV SS, MSEL *T* scores, and CBCL *T* scores

Measure	M (SD)	Mdn (IQR)	Range^a^
Sleep latency (min)	35.35 (21.56)	30 (20–45)	5–90
Nighttime sleep duration (hr)	10.36 (1.09)	10.5 (9.8–11)	7.3–13.3
CDI-EV SS	99.50 (11.04)	98 (90.5–108)	84–130
MSEL *T* scores			
MSEL Expressive Language *T*	32.20 (10.08)	30 (22.3–38)	20–58
MSEL Receptive Language *T*	31.93 (11.66)	31.5 (20–41)	20–56
MSEL Visual Reception *T*	31.69 (10.16)	31 (20–40)	20–56
CBCL DSM-5-based scales			
Depressive Problems *T*	58.01 (6.78)	56 (52–62.3)	50–77
Anxiety Problems *T*	51.38 (2.69)	50 (50–51)	50–60
Autism Spectrum Problems *T*	56.32 (7.27)	54 (51–61)	50–87
Attention/Hyperactivity Problems *T*	55.93 (6.72)	54 (50–60)	50–76
Oppositional Defiant Problems *T*	52.16 (4.77)	50 (50–52)	50–77
CBCL 2007 Scale			
Stress Problems *T*	54.29 (6.27)	51 (51–56.8)	50–85
CBCL Higher-order Factor Scales			
Internalizing Problems *T*	50.63 (8.14)	49 (45–57.5)	29–68
Externalizing Problems *T*	49.63 (9.81)	49 (42–55.8)	32–80
Total Problems *T*	51.42 (8.65)	52 (44–57)	31–73

*CDI-EV SS* MacArthur-Bates Communicative Development Inventory: Words and Sentences expressive vocabulary standard score, *MSEL* Mullen Scales of Early Learning, *CBCL* Child Behavior Checklist, *IQR* interquartile range, *min* minutes, *hr* hours, *DSM-5* Diagnostic and Statistical Manual of Mental Disorders-5

^a^Lowest possible score: CDI-EV SS at 24 months = 84; MSEL *T* score = 20

**Fig. 1:**
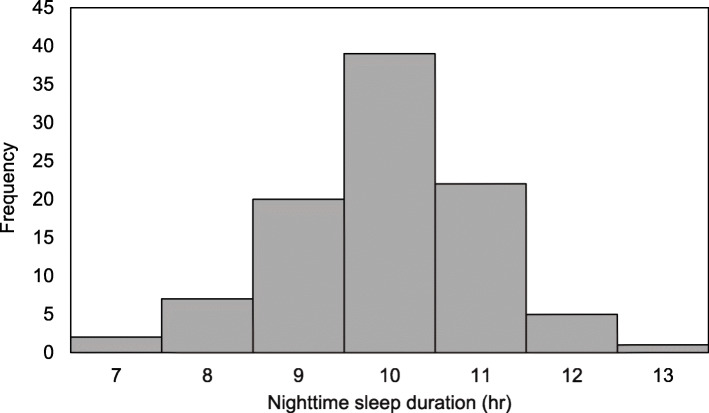
Distribution of parent-reported nighttime sleep duration (in hours) for 96 2-year-olds with Williams syndrome

As shown in Fig. 1, nighttime sleep duration was normally distributed. As indicated in Table 2, mean nighttime sleep duration was 10.36 ± 1.1 h, which did not differ significantly from that reported by Bell and Zimmerman [55] for 822 TD toddlers (10.4 ± 1.4 h), *t*(916) = − 0.270, *p* = .787. Children took a median of 30 min to fall asleep, with 23% (*n* = 22) of the sample taking more than 45 min.

To provide further descriptive information regarding sleep for 2-year-olds with WS, parent responses to several other questions on the PSQ were considered. Parents of 57 of the 96 children (59.4%) indicated that their child had difficulty falling asleep. Thirteen children (out of 95, 13.7%) were reported to wake up more than twice per night and 26 (out of 95, 27.4%) had difficulty falling back asleep if they woke up at night. Parents of 27 (out of 91, 29.7%) indicated that their child had restless sleep and parents of 17 (out of 95, 17.9%) indicated their child had a difficult bedtime routine. Thirteen children (out of 96, 13.5%) took medication for sleep: 12 (12.5%) took melatonin (one of whom also took Clonazepam and another of whom also took Periactin, an anti-histamine) and one took only Periactin. Not surprisingly given the participants’ ages, 85 (out of 95, 89.5%) took a daytime nap.

Descriptive statistics for the sleep, language, cognitive, and behavioral measures for the full sample are provided in Table 2. Sleep latency, nighttime sleep duration, SRBD score, and Sleepiness score were not significantly related to CDI-EV SS or to the child’s age; the absolute value of these correlations ranged from .01 (*p* = .945) to .20 (*p* = .100). As indicated in Table 3, sleep latency was significantly negatively correlated with nighttime sleep duration, and SRBD score was significantly negatively related to MSEL Receptive Language *T*. None of the other correlations between the sleep variables and MSEL *T* scores was significant. Few significant relations were found between either sleep latency or nighttime sleep duration and CBCL *T* scores. In contrast, most of the correlations between SRBD or Sleepiness scores and CBCL *T* scores were statistically significant.

**Table 3:** Bivariate correlations among sleep measures, CBCL *T* scores, and MSEL *T* scores

	Sleep latency	Nighttime sleep duration	SRBD score	Sleepiness score
CBCL Depressive Problems *T*	.34*	− .31*	.63**	.52**
CBCL Anxiety Problems *T*	.04	− .27*	.18	− .06
CBCL Autism Spectrum Problems *T*	.04	− .08	.31*	.15
CBCL Attention/Hyperactivity Problems *T*	.01	− .19	.58**	.50**
CBCL Oppositional Defiant Problems *T*	− .15	.12	.35*	.32*
CBCL Stress Problems *T*	.05	− .08	.48**	.37*
CBCL Sleep Problems *T*	.34*	− .30*	.51**	.46**
CBCL Internalizing Problems *T*	.08	− .22	.45**	.17
CBCL Externalizing Problems *T*	.03	− .02	.55**	.50**
CBCL Total Problems *T*	.18	− .20	.67**	.50**
MSEL Expressive Language *T*	< − .01	.17	− .18	− .10
MSEL Receptive Language *T*	− .06	.16	− .29*	− .15
MSEL Visual Reception *T*	.07	− .08	− .26	− .19
Sleep latency		− .41**	.20	.05
Nighttime sleep duration			− .21	− .01
SRBD score				---
Sleepiness score				

*N* = 96 for all correlations except those including Sleepiness score, for which *N* = 70

*CBCL* Child Behavior Checklist, *MSEL* Mullen Scales of Early Learning, *SRBD* Sleep-Related Breathing Disorders

^*^p < .01, **p < .001

### Comparison of children who did or did not screen positive on the PSQ SRBD scale and subscales

Descriptive statistics for sleep latency, nighttime sleep duration, and CBCL *T* scores are reported in Table 4, separately for children who did/did not screen positive for the SRBD scale and children who did/did not screen positive for the Sleepiness subscale. As indicated in the table, there were no significant differences between those who screened positive compared to those who did not for either sleep latency or nighttime sleep duration. In contrast, the SRBD-positive screen group evidenced significantly more difficulties than the SRBD-negative screen group on all of the CBCL scales and higher-order factors. Those who screened positive on the Sleepiness scale demonstrated significantly more problems on the Attention Deficit/Hyperactivity and Stress scales and the Externalizing and Total factor scales than those who screened negative.

**Table 4:** Descriptive statistics for sleep latency, nighttime sleep duration, and CBCL *T* scores as a function of positive/negative screen for the Pediatric Sleep Questionnaire SRBD scale and Sleepiness subscale

Measure	*N*	*M* (SD)	Mdn (IQR)	Range	Mann-Whitney *U* (positive vs. negative)		
					*Z*	*p*	Cohen’s *d*
Sleep latency (min)							
SRBD							
Positive	15	40.00 (20.96)	30 (30–60)	15–90	1.11	.266	0.23
Negative	81	34.49 (21.69)	30 (18.5–45.0)	5–90			
Sleepiness							
Positive	21	34.38 (17.51)	30 (17.5–48.5)	10–60	0.70	.486	0.08
Negative	49	32.82 (21.86)	30 (16–45)	10–90			
Nighttime sleep duration (hr)							
SRBD							
Positive	15	10.05 (1.29)	10.5 (9–11)	7.5–12.3	0.83	.405	0.17
Negative	81	10.42 (1.05)	10.5 (9.8–11)	7.3–13.3			
Sleepiness							
Positive	21	10.50 (1.22)	10.5 (9.6–11.4)	8.3–13.3	0.07	.944	0.01
Negative	49	10.51 (0.94)	10.5 (10–11)	8.3–12.5			
CBCL DSM-5-based scales							
Depressive Problems *T*							
SRBD							
Positive	15	67.73 (6.02)	67 (63–72)	56–77	5.13	< .001*	1.21
Negative	81	56.21 (5.22)	56 (51–60)	50–70			
Sleepiness							
Positive	21	61.29 (8.44)	60 (54–67)	50–77	2.45	.014	0.86
Negative	49	55.90 (5.13)	56 (51–60)	50–67			
Anxiety Problems *T*							
SRBD							
Positive	15	52.60 (3.96)	51 (50–54)	50–60	2.13	.033*	0.37
Negative	81	51.15 (2.35)	50 (50–51)	50–60			
Sleepiness							
Positive	21	50.90 (2.28)	50 (50–51)	50–60	0.20	.843	0.05
Negative	49	51.02 (2.29)	50 (50–51)	50–60			
Autism Spectrum Problems *T*							
SRBD							
Positive	15	59.47 (7.38)	58 (54–61)	51–79	2.63	.016*	0.50
Negative	81	55.74 (7.14)	54 (51–58)	50–87			
Sleepiness							
Positive	21	58.05 (8.66)	58 (51–61)	50–87	0.76	.448	0.27
Negative	49	56.12 (6.44)	54 (51–61)	50–70			
Attention/Hyperactivity Problems *T*							
SRBD							
Positive	15	62.87 (6.42)	64 (57–67)	54–76	4.27	< .001*	0.95
Negative	81	54.64 (5.97)	52 (50–51)	50–76			
Sleepiness							
Positive	21	60.71 (7.04)	60 (55.5–65.5)	51–76	4.50	< .001*	1.21
Negative	49	53.41 (5.53)	51 (50–54)	50–76			
Oppositional Defiant Problems *T*							
SRBD							
Positive	15	56.80 (9.07)	52 (50–64)	50–77	2.77	.006*	0.53
Negative	81	51.30 (2.81)	50 (50–51)	50–64			
Sleepiness							
Positive	21	54.33 (6.77)	51 (50–57)	50–70	2.02	.043	0.79
Negative	49	51.12 (2.12)	50 (50–52)	50–59			
CBCL 2007 Scale							
Stress Problems *T*							
SRBD							
Positive	15	61.27 (10.41)	63 (53–67)	51–85	3.87	< .001*	0.83
Negative	81	53.00 (4.12)	51 (50–53)	50–70			
Sleepiness							
Positive	21	56.81 (8.61)	53 (51–60.5)	50–85	2.87	.004*	0.66
Negative	49	52.92 (4.37)	51 (50–53)	50–70			
CBCL Higher-order Factor Scales							
Internalizing Problems *T*							
SRBD							
Positive	15	56.80 (6.39)	59 (51–60)	45–68	3.14	.002*	0.67
Negative	81	49.48 (7.94)	47 (45–55.5)	29–66			
Sleepiness							
Positive	21	51.43 (8.77)	51 (47–58.5)	33–68	1.04	.300	0.21
Negative	49	49.67 (8.04)	47 (45–56)	29–64			
Externalizing Problems *T*							
SRBD							
Positive	15	61.20 (9.40)	58 (54–65)	47–80	4.51	< .001*	1.03
Negative	81	47.48 (8.31)	46 (40–54)	31–67			
Sleepiness							
Positive	21	57.00 (9.92)	56 (50–61)	39–80	4.09	< .001*	1.22
Negative	49	46.49 (7.97)	46 (40–51.5)	32–64			
Total Problems *T*							
SRBD							
Positive	15	62.67 (5.82)	62 (57–66)	55–73	5.19	< .001*	1.25
Negative	81	49.33 (7.40)	50 (43–55)	31–65			
Sleepiness							
Positive	21	56.81 (8.07)	57 (53–61)	40–73	3.81	< .001*	1.13
Negative	49	48.33 (7.30)	50 (43–53.5)	31–65			

*CBCL* Child Behavior Checklist, *SRBD* Sleep-Related Breathing Disorder, *IQR* interquartile range, *min* minutes, *hr* hours, *DSM-5* Diagnostic and Statistical Manual of Mental Disorders-5

*Difference remains statistically significant after Holm-Bonferroni correction

Descriptive statistics for performance on the language and nonverbal reasoning measures as a function of positive/negative screen for SRBD and for Sleepiness are provided in Table S1. As indicated in the table, after Holm-Bonferroni corrections, there were no significant differences on any of these measures between children with or without a positive screen on either the SRBD scale or the Sleepiness subscale.

We also compared the sleep latency and nighttime sleep duration of children with positive and negative screens on the SRBD Snoring subscale and the SRBD Behavior subscale. For the Snoring subscale, those who screened positive took significantly longer to fall sleep (*N* = 26, *Mdn* = 42.50, *M* = 45.35, *SD* = 23.16) than those who screened negative (*N* = 66,* Mdn* = 30.00, *M* = 31.29, *SD* = 19.83), *Z* = 2.81, *p* = .005, Cohen’s *d* = 0.61. Similarly, for the Behavior subscale, children with a positive screen took significantly longer to fall sleep (*N* = 75, *Md*n = 30.00, *M* = 37.32, *SD *= 20.64) than those with a negative screen (*N* = 21, *Mdn* = 20.00, *M* = 28.33, *SD* = 23.78), *Z* = 2.42, *p* = .016, Cohen’s *d* = 0.51. No significant differences were found for nighttime sleep duration or any of the language or nonverbal reasoning measures between groups that screened positive or negative on either the Snoring or Behavior subscales.

For the PSQ Behavior subscale, those with a positive screen had significantly more difficulty than those who screened negative on the Stress (positive: *Mdn* = 53.00, *M* = 54.91, *SD* = 6.64; negative: *Mdn* = 50.00, *M* = 52.10, *SD* = 4.17; *Z* = 3.11, *p* = .002, Cohen’s *d* = 0.65) and Internalizing scales (positive: *Mdn* = 51.00, *M* = 51.87, *SD* = 7.71; negative: *Mdn* = 47.00, *M* = 46.38, *SD* = 8.21; *Z* = 2.53, *p* = .011, Cohen’s *d* = 0.53). Due to the overlap in items between the PSQ Behavior subscale and the CBCL Attention Deficit/Hyperactivity Problems scale and the Externalizing factor scale, comparisons between positive and negative screen groups were not performed. No significant differences were found on the remaining CBCL scales. For the Snoring subscale, there were no significant differences between those who screened positive and those who screened negative on any of the CBCL scales.

### Correlations among variables to be included in the regression analyses

Bivariate correlations among the variables included in the regression analyses are reported in Table 5. As indicated in the table, all correlations among language and cognitive measures were positive and significant. All of the language measures were positively correlated with nighttime sleep duration and negatively correlated with Sleepiness score, although none of these correlations was strong enough to reach the *p* = .01 level of statistical significance. CBCL Internalizing Problems *T* was significantly negatively correlated with all language and nonverbal reasoning measures and significantly positively correlated with CBCL Externalizing Problems *T*, which was significantly positively correlated with Sleepiness score.

**Table 5:** Bivariate correlations among the measures included in the regression analyses

	2	3	4	5	6	7	8
1. CDI-EV SS	.88**	.65**	.63**	− .31*	.01	.17	− .09
2. MSEL Expressive Language *T*		.68**	.63**	− .28*	− .05	.17	− .10
3. MSEL Receptive Language *T*			.68**	− .32*	− .05	.16	− .15
4. MSEL Visual Reception *T*				− .36**	− .13	− .08	− .19
5. CBCL Internalizing Problems *T*					.45**	− .22	.17
6. CBCL Externalizing Problems *T*						− .02	.50**
7. Nighttime sleep duration							− .01
8. Sleepiness score							

*N* = 96 for all correlations except those including Sleepiness score, for which *N* = 70

*CDI-EV SS* MacArthur-Bates Communicative Development Inventory: Words and Sentences expressive vocabulary standard score, *MSEL* Mullen Scales of Early Learning, *CBCL* Child Behavior Checklist

^*^p < .01, **p < .001

### Association of language ability and behavior problems with nonverbal reasoning ability and nighttime sleep duration

To better understand the concurrent contributions of nonverbal reasoning ability and nighttime sleep duration to language abilities and behavior problems, five linear multiple regressions were conducted. As indicated in Table 6, for the three models that included language as the outcome, MSEL Visual Reception *T* (large effect) and nighttime sleep duration (small effect) made significant independent contributions and together explained a large amount of the variance, with Visual Reception *T* uniquely accounting for between 41 and 49% and nighttime sleep duration uniquely accounting for between 4.8 and 5.3%.

As indicated in Table 6, the fourth model explained a medium amount of the variance in CBCL Internalizing Problems *T*, with significant independent contributions from MSEL Visual Reception *T* (medium effect) and nighttime sleep duration (small effect). Visual Reception *T* uniquely accounted for 14.4% of the variance and nighttime sleep duration uniquely accounted for 6.3%. The fifth model did not account for a significant amount of variance in CBCL Externalizing Problems *T*.

### Association of language ability and behavior problems with nonverbal reasoning ability and excessive daytime sleepiness

To investigate the concurrent contributions of nonverbal reasoning ability and excessive daytime sleepiness to language abilities and behavior problems, five linear multiple regressions were conducted. As indicated in Table 7, the first three models explained a large amount of the variance in each of the three measures of language abilities. MSEL Visual Reception *T* (large effect) was the only significant concurrent predictor, uniquely accounting for between 34.8 and 50.4% of the variance. The fourth model explained a medium amount of the variance in CBCL Internalizing Problems *T*. MSEL Visual Reception *T* (small effect) was the only significant concurrent predictor, uniquely accounting for 12.3% of the variance. The fifth model explained a large amount of the variance in CBCL Externalizing Problems *T*. Sleepiness score (medium effect) was the only significant concurrent predictor, uniquely accounting for 22.1% of the variance (Table 7).

**Table 6:** Multiple regression analyses predicting concurrent language and behavior as a function of nonverbal reasoning and nighttime sleep duration

Predictor	*B*	*t*	*p* -value	95% CI for *B*	Semi-partial *r*	Cohen’s *f*^2^
1. CDI-EV SS						
Constant	99.50	116.91	< .001	[97.81, 101.19]		
MSEL Visual Reception *T*	0.70	8.27	< .001*	[0.53, 0.87]	.64	0.74
Nighttime sleep duration (hr)	2.30	2.92	.004*	[0.74, 3.86]	.23	0.09
*R*^2^ = .44, adjusted *R*^2^ = .43, *F* (2, 93) = 36.75, *p* < .001						
2. MSEL Expressive Language *T*						
Constant	32.19	41.49	< .001	[30.65, 33.74]		
MSEL Visual Reception *T*	0.64	8.33	< .001*	[0.49, 0.80]	.65	0.75
Nighttime sleep duration (hr)	2.02	2.82	.006*	[0.60, 3.45]	.22	0.08
*R*^2^ = .44, adjusted *R*^2^ = .43, *F* (2, 93) = 37.00, *p* < .001						
3. MSEL Receptive Language *T*						
Constant	31.92	38.00	< .001	[30.25, 33.59]		
MSEL Visual Reception *T*	0.81	9.64	< .001*	[0.64, 0.97]	.70	1.00
Nighttime sleep duration (hr)	2.31	2.97	.004*	[0.77, 3.85]	.22	0.09
*R*^2^ = .51, adjusted *R*^2^ = .50, *F* (2, 93) = 48.88, *p* < .001						
4. CBCL Internalizing Problems *T*						
Constant	50.67	67.45	< .001	[49.18, 52.16]		
MSEL Visual Reception *T*	− 0.31	− 4.11	< .001*	[− 0.46, − 0.16]	− .38	0.18
Nighttime sleep duration (hr)	− 1.84	− 2.65	.010*	[− 3.22, − 0.46]	− .25	0.08
*R*^2^ = .19, adjusted *R*^2^ = .18, *F* (2, 93) = 11.14, *p* < .001						
5. CBCL Externalizing Problems *T*						
Constant	49.65	49.56	< .001	[47.66, 51.64]		
MSEL Visual Reception *T*	− 0.12	− 1.25	.216	[− 0.32, 0.07]	− .13	0.02
Nighttime sleep duration (hr)	− 0.28	− 0.30	.763	[− 2.12, 1.56]	− .03	< 0.01
*R*^2^ = .02, adjusted *R*^2^ = − .004, *F* (2, 93) = .80, *p* = .454						

*N* = 96*. CI* confidence interval, *CDI-EV SS* MacArthur-Bates Communicative Development Inventory: Words and Sentences expressive vocabulary standard score, *MSEL* Mullen Scales of Early Learning, *hr* hours, *CBCL* Child Behavior Checklist

*Effect remains statistically significant after Holm-Bonferroni correction

**Table 7:** Multiple regression analyses predicting concurrent language and behavior as a function of nonverbal reasoning and sleepiness

Predictor	*B*	*t*	*p* -value	95% CI for *B*	Semi-partial *r*	Cohen’s *f*^2^
1. CDI-EV SS						
Constant	99.84	94.96	< .001	[97.74, 101.93]		
MSEL Visual Reception *T*	0.66	5.96	< .001*	[0.44, 0.88]	.59	0.53
Sleepiness score	0.28	0.24	.809	[− 2.03, 2.59]	.02	− 0.06
*R*^2^ = .35, adjusted *R*^2^ = .33, *F* (2, 67) = 18.16, *p* < .001						
2. MSEL Expressive Language *T*						
Constant	32.00	36.37	< .001	[30.25, 33.76]		
MSEL Visual Reception *T*	0.65	7.08	< .001*	[0.47, 0.84]	.65	0.75
Sleepiness score	0.25	0.26	.795	[− 1.68, 2.19]	.02	0.07
*R*^2^ = .43, adjusted *R*^2^ = .42, *F* (2, 67) = 25.60, *p* < .001						
3. MSEL Receptive Language *T*						
Constant	32.31	31.82	< .001	[30.29, 34.34]		
MSEL Visual Reception *T*	0.90	8.42	< .001*	[0.69, 1.11]	.71	1.06
Sleepiness score	− 0.24	− 0.22	.829	[− 2.48, 1.99]	− .02	0.12
*R*^2^ = .53, adjusted *R*^2^ = .51, *F* (2, 67) = 37.04, *p* < .001						
4. CBCL Internalizing Problems *T*						
Constant	50.01	54.15	< .001	[48.17, 51.86]		
MSEL Visual Reception *T*	− 0.30	− 3.07	.003*	[− 0.49, − 0.10]	− .35	0.14
Sleepiness score	0.93	0.91	.364	[− 1.10, 2.96]	.10	0.01
*R*^2^ = .15, adjusted *R*^2^ = .12, *F* (2, 67) = 5.86, *p* = .005						
5. CBCL Externalizing Problems *T*						
Constant	49.56	48.26	< .001	[47.51, 51.61]		
MSEL Visual Reception *T*	− 0.10	− 0.89	.377	[− 0.31, 0.12]	− .09	0.01
Sleepiness score	5.09	4.50	< .001*	[2.83, 7.35]	.47	0.30
*R*^2^ = .26, adjusted *R*^2^ = .24, *F* (2, 67) = 11.66, *p* < .001						

*N* = 70. *CI* confidence interval, *CDI-EV SS* MacArthur-Bates Communicative Development Inventory: Words and Sentences expressive vocabulary standard score, *MSEL* Mullen Scales of Early Learning, *CBCL* Child Behavior Checklist

*Effect remains statistically significant after Holm-Bonferroni correction

## Discussion

In this study, we determined the parent-reported sleep characteristics and problems of a relatively large sample of 2-year-olds with WS and considered whether children who screened positive for SRBD or components of SRBD had more limited language and nonverbal reasoning abilities and more behavior problems than those who screened negative. We also considered potential concurrent contributions of nonverbal reasoning ability, nighttime sleep duration, and excessive daytime sleepiness to expressive language, receptive language, and behavior problems.

### Sleep characteristics and problems in toddlers with Williams syndrome

Nighttime sleep duration for the participants in this study did not differ significantly from same-aged TD children from Bell and Zimmerman’s [55] previous study. This finding is consistent with the findings from the prior study of infants with WS [10] and about half of the previous studies for individuals with WS beyond infancy [42–44, 46] (but see [11, 47]). As reported in previous studies ([41–43, 47] but see [10]), sleep latency was quite long for many participants. Many participants had restless sleep (previously reported by [41, 46]) and/or had difficulty falling back asleep if they woke up at night (previously reported by [41, 42]). The majority evidenced attention difficulties as measured by the PSQ Behavior scale, consistent with prior findings for young children with WS on both the CBCL [60] and DSM-IV-based diagnostic interviews with parents [22].

Our results suggest that the rate of SRBD for toddlers with WS is higher than that for toddlers in the general population [33]. The finding that a third of our sample evidenced excessive daytime sleepiness problems and almost a third snored is consistent with results for adolescents and adults with WS [44]. These results also are consistent with previous reports [6, 8] that sleep disturbances are highly prevalent in children with neurodevelopmental disorders relative to TD children.

### Comparison of children who did or did not screen positive on the PSQ SRBD scale and subscales

After Holm-Bonferroni correction, we did not find significant differences between the toddlers with WS who screened positive on the PSQ SRBD scale or subscales and the toddlers who screened negative on any of the language or nonverbal reasoning variables. The lack of significant differences may reflect a lack of power due to the small sample sizes for children who screened positive on the SRBD scale or subscales.

Consistent with prior findings for TD children [34], 2-year-olds with WS who screened positive for SRBD had significantly more behavior problems than those who screened negative. We found large effect sizes for depressive problems, attention deficit/ hyperactivity problems, stress problems, and externalizing problems overall and a medium effect size for internalizing problems. A similar pattern of effects was found for those who screened positive for excessive daytime sleepiness, even if they did not screen positive for SRBD. This pattern is similar to that reported by O’Brien et al. [26], who found that sleepiness rather than SRBD was driving the relations between sleep problems and aggressive behavior for TD school-aged children. These results highlight the importance of assessing sleep problems such as SRBD and excessive daytime sleepiness early in life in children with neurodevelopmental disorders since these problems may underlie and/or exacerbate observed behavior problems.

### Nonverbal reasoning ability, nighttime sleep duration, and excessive daytime sleepiness as concurrent contributors to language ability and behavior problems

Previous studies of TD preschoolers [4, 61] and children with Down syndrome [11] have demonstrated a significant relation between nighttime sleep duration and vocabulary development. The prior small-sample studies of young children with WS [11, 47], both of which included children varying in age by several years, also suggested a possible relation, although the results for expressive vocabulary were mixed.

In the present study, we found significant concurrent relations between nighttime sleep duration and both expressive language and receptive language—even after controlling for nonverbal reasoning ability—for a considerably larger sample of children over a very narrow age range. Importantly, the present study extended prior findings for young children with WS by demonstrating relations not only with parent report measures of vocabulary but also with direct assessments of children’s expressive and receptive language, even after accounting for nonverbal reasoning ability, a major potentially confounding variable that was not considered in previous studies. The effect size for nighttime sleep duration on early language development has consistently been small. In contrast, the effect size for nonverbal reasoning ability was large for each of the language measures considered in the present study. This pattern suggests that in future studies of the impact of sleep variables on language ability, nonverbal reasoning ability also should be assessed.

This is the first study to demonstrate significant concurrent relations between sleep variables and behavior problems in children with WS. In particular, similar to previous findings for TD children [34], nighttime sleep duration had a small but significant concurrent relation with internalizing behavior problems in 2-year-olds with WS. Shorter nighttime sleep duration was significantly associated with increased internalizing problems even after accounting for nonverbal reasoning ability, which had a medium effect. The concurrent relation between nighttime sleep duration and externalizing behavior problems was not statistically significant. Daytime sleepiness did not have a significant concurrent relation with language ability or internalizing problems for 2-year-olds with WS. However, consistent with prior findings for TD children [26, 27], daytime sleepiness was a significant concurrent contributor to externalizing problems. Higher levels of daytime sleepiness were significantly associated with increased externalizing problems, even after accounting for nonverbal reasoning ability, which did not have a significant effect.

### Limitations

The results of the present study should be interpreted in the context of certain limitations. First, this study was not sufficiently powered to conclusively determine if the nonverbal reasoning or language abilities of 2-year-olds with WS who screened positive for SRBD were more limited than those who screened negative. This is because only 15 of 96 children met criteria for SRBD. However, the effects of SRBD on behavior were so pervasive that we found significant and large differences between the SRBD screen-positive and screen-negative groups despite the small size of the screen-positive group.

Another limitation is that our measure of nighttime sleep duration is almost certainly an overestimate of the amount of time the participants were asleep between when they first fell asleep at night and when they woke up in the morning. This is because our measure did not take into account the duration of any nighttime awakenings. More generally, parent report has been shown to overestimate sleep duration compared to actigraphy [62]. In future studies, this limitation would be best addressed by using actigraphy in conjunction with parental report [62]. Although sleep diaries would document some nighttime awakenings, individual differences in children in whether or how loudly they vocalized or cried after waking up at night and individual differences among parents in how much noise was necessary to awaken them would affect parents’ detection of nighttime awakenings, introducing unintended sources of variability.

It also is possible that some of the shared variance between sleep variables as assessed by the PSQ and expressive language as measured by the CDI or behavior as measured by the CBCL is due to common-method variance, as the PSQ, CDI, and CBCL are all parent report measures. In this context, it is important to note that the amount of unique variance explained by the sleep variables in expressive language as measured by parent report on the CDI and by performance on the examiner-administered MSEL Expressive Language scale was highly similar. In addition, Gwilliam et al. [63] recently reported a very strong correlation between parent-reported and actigraphy-based nighttime sleep duration for a small sample of 24-month-olds with WS. These findings reduce the likelihood that the significant findings in the present study are due primarily to common-method variance.

Finally, the cross-sectional nature and correlational design of the present study do not allow us to draw conclusions about causality. Longitudinal studies are needed to identify the pathways through which early sleep problems and nighttime sleep duration may affect later language development and/or behavior.

## Conclusions

The prevalence of positive screens for SRBD and other sleep problems among 2-year-olds with WS is higher than expected for children in the general population. Furthermore, 2-year-olds with WS who screened positive for SRBD evidenced significantly more difficulties with internalizing behavior, externalizing behavior, and stress, and those who screened positive for excessive daytime sleepiness evidenced significantly more difficulties with externalizing behavior and stress than did those who screened negative. Screening for SRBD, excessive daytime sleepiness, and other sleep problems should be standard practice when evaluating children with WS, and more generally, young children with neurodevelopmental disorders. These findings also support the need for studies to determine whether targeted early intervention for SRBD or its components for young children with WS or more generally for young children with neurodevelopmental disorders would reduce the rate or level of early behavior problems.

For very young children with WS, nighttime sleep duration is positively associated with language development—as measured by both parent report and examiner-administered standardized assessment—after taking into account the contribution of nonverbal reasoning ability. In addition, nighttime sleep duration is negatively associated with parent-reported internalizing behavior problems, and excessive daytime sleepiness is positively associated with parent-reported externalizing problems, after taking into account the children’s nonverbal reasoning ability. These patterns parallel prior findings for TD toddlers. Provision of guidance regarding good sleep hygiene by pediatricians and early intervention specialists and support in implementing these guidelines has the potential to improve developmental outcomes. It is important that behavioral strategies such as a consistent sleep-wake schedule and a calming bedtime routine that maximizes the likelihood that the child will be able to self-calm after awakening during the night be offered to parents of young children with WS in both oral and written form. These strategies have been shown to increase nighttime sleep duration both in young TD children [64] and, when provided with appropriate modifications, in children with neurodevelopmental disorders [65]. Ongoing support, including tracking of implementation and effectiveness, will almost certainly be needed. Results of intervention studies including children with WS that focus on these types of behavioral strategies with or without medications such as melatonin could lead to recommendations targeted to maximize developmental outcomes for children with this syndrome.

## Supplementary Information

Additional file 1: **Table S1.** Descriptive statistics for CDI expressive vocabulary standard scores and Mullen Scales of Early Learning *T*-scores as a function of positive/negative screen on the Pediatric Sleep Questionnaire SRBD scale and Sleepiness subscale.

## Data Availability

The data analyzed for this paper can be made available upon a reasonable request to the corresponding author.

## Abbreviations

CBCL: Child Behavior Checklist
CDI: MacArthur-Bates Communicative Development Inventory: Words and Sentences
CI: Confidence interval
DSM: Diagnostic and Statistical Manual of Mental Disorders
EV: Expressive vocabulary
IQR: Interquartile range
MSEL: Mullen Scales of Early Learning
PSQ: Pediatric Sleep Questionnaire
SRBD: Sleep-Related Breathing Disorder
SS: Standard score
TD: Typically developing
WS: Williams syndrome
