# Supplementary material for: Sleep characteristics and problems of 2-year-olds with Williams syndrome: relations with language and behavior
Source: J Neurodev Disord. 2020 Nov 20;12:32. doi: 10.1186/s11689-020-09336-z (PMC7679988; doi:10.1186/s11689-020-09336-z)
Supplement: Supplementary file 1 — Table S1. Descriptive statistics for CDI expressive vocabulary standard scores and Mullen Scales of Early Learning T-scores as a function of positive/negative screen on the Pediatric Sleep Questionnaire SRBD scale and Sleepiness subscale. [file 11689_2020_9336_MOESM1_ESM.pdf]

Table S1 Descriptive statistics for CDI expressive vocabulary standard scores and Mullen Scales of Early Learning T-scores as a function of positive/negative screen for the Pediatric Sleep Questionnaire SRBD scale and Sleepiness subscale

					Mann-Whitney *U* (Positive *vs*. Negative)		
Measure	N	M (SD)	Mdn (IQR)	Range[a]	*Z*	*p*	Cohen's *d*
CDI-EV SS							
SRBD: Positive	15	96.93 (9.00)	96 (90 – 100)	84 – 120	0.93	.352	0.19
Negative	81	99.98 (11.36)	98 (91 – 108)	84 – 130			
Sleepiness: Positive	21	99.43 (11.68)	96 (92 – 106)	84 – 130	0.25	.802	< 0.01
Negative	49	99.43 (10.44)	98 (90 – 108)	84 – 124			
MSEL Expressive Language *T*							
SRBD: Positive	15	29.53 (6.81)	29 (26 – 32)	20 – 46	1.00	.317	0.20
Negative	81	32.69 (10.54)	32 (21.5 – 39.5)	20 – 58			
Sleepiness: Positive	21	31.24 (9.96)	30 (22.5 – 35.5)	20 – 58	0.30	.762	0.05
Negative	49	31.76 (9.57)	32 (20.5 – 37.5)	20 – 53			
MSEL Receptive Language *T*							
SRBD: Positive	15	25.40 (8.30)	20 (20 – 32)	20 – 32	2.27	.023[b]	0.47
Negative	81	33.14 (11.83)	33 (20 – 43)	20 – 56			
Sleepiness: Positive	21	28.71 (11.04)	24 (20 – 37.5)	20 – 54	1.30	.193	0.36
Negative	49	33.06 (12.44)	31 (20 – 43.5)	20 – 56			
MSEL Visual Reception *T*							
SRBD: Positive	15	26.07 (8.56)	20 (20 – 34)	20 – 43	2.45	.014[b]	0.51
Negative	81	32.73 (10.13)	32 (24 – 40)	20 – 56			
Sleepiness: Positive	21	29.43 (10.09)	28 (20 – 38)	20 – 53	1.05	.293	0.24
Negative	49	31.78 (9.59)	31 (24 – 39)	20 – 55			

Note. CDI = MacArthur-Bates Communicative Development Inventory: Words and Sentences; EV = expressive vocabulary; SS = standard score; MSEL = Mullen Scales of Early Learning; IQR = interquartile range; SRBD = Sleep-Related Breathing Disorders.
[a]Lowest possible CDI-EV SS at 24 months = 84; lowest possible MSEL T-score = 20.
[b]Difference is not statistically significant after Holm-Bonferroni correction.
